# Biological Characterization of Yellow Fever Viruses Isolated From Non-human Primates in Brazil With Distinct Genomic Landscapes

**DOI:** 10.3389/fmicb.2022.757084

**Published:** 2022-02-14

**Authors:** Nathália Dias Furtado, Lidiane de Menezes Raphael, Ieda Pereira Ribeiro, Iasmim Silva de Mello, Déberli Ruiz Fernandes, Mariela Martínez Gómez, Alexandre Araújo Cunha dos Santos, Mônica da Silva Nogueira, Márcia Gonçalves de Castro, Filipe Vieira Santos de Abreu, Lívia Carício Martins, Pedro Fernando da Costa Vasconcelos, Ricardo Lourenço-de-Oliveira, Myrna Cristina Bonaldo

**Affiliations:** ^1^Laboratório de Biologia Molecular de Flavivírus, Instituto Oswaldo Cruz/Fundação Oswaldo Cruz (FIOCRUZ), Rio de Janeiro, Brazil; ^2^Instituto de Investigaciones Biológicas Clemente Estable, Montevideo, Uruguay; ^3^Centro de Experimentação Animal, Instituto Oswaldo Cruz/Fundação Oswaldo Cruz (FIOCRUZ), Rio de Janeiro, Brazil; ^4^Laboratório de Mosquitos Transmissores de Hematozoários, Instituto Oswaldo Cruz/Fundação Oswaldo Cruz (FIOCRUZ), Rio de Janeiro, Brazil; ^5^Seção de Arbovirologia e Febres Hemorrágicas, Instituto Evandro Chagas/Fundação Oswaldo Cruz (FIOCRUZ), Pará, Brazil

**Keywords:** yellow fever virus, Brazil outbreaks, genetic markers, virulence, mouse neurovirulence, cell infection, interferon-mediated viral inhibition

## Abstract

Since the beginning of the XXI Century, the yellow fever virus (YFV) has been cyclically spreading from the Amazon basin to Brazil’s South and Southeast regions, culminating in an unprecedented outbreak that started in 2016. In this work, we studied four YFV isolated from non-human primates obtained during outbreaks in the states of Rio Grande do Sul in 2008 (PR4408), Goiás (GO05), and Espírito Santo (ES-504) in 2017, and Rio de Janeiro (RJ 155) in 2019. These isolates have genomic differences mainly distributed in non-structural proteins. We compared the isolates’ rates of infection in mammal and mosquito cells and neurovirulence in adult mice. RJ 155 and PR4408 YFV isolates exhibited higher infectivity in mammalian cells and neurovirulence in mice. In mosquito Aag2 cells, GO05 and PR4408 displayed the lowest proliferation rates. These results suggest that RJ 155 and PR4408 YFV isolates carry some genomic markers that increase infectivity in mammal hosts. From this characterization, it is possible to contribute to discovering new molecular markers for the virulence of YFV.

## Introduction

Yellow fever virus (YFV) is the prototype member of the genus *Flavivirus* (family *Flaviviridae*), which comprises other arthropod-borne viruses of clinical relevance. It is a small, enveloped virus with a genome consisting of a positive-sense single-stranded RNA of about 11 kb, encoding a single polyprotein flanked by two untranslated regions (UTR) (5′UTR and 3′UTR). This polyprotein is processed co- and post-translationally generating three structural proteins: capsid (C), membrane (prM/M), and envelope (E), that hold the outer structure and mediate the initial host/virus interaction; and seven non-structural (NS) proteins: NS1, NS2A, NS2B, NS3, NS4A, NS4B, and NS5, which play essential roles on genome replication and immune evasion (Marfin and Monath, 2008).

Infection by YFV causes a febrile disease whose symptoms range from mild to severe, more frequently with liver, spleen, and kidney damages. The fatality rate of individuals presenting severe symptoms ranges between 20 and 50% (Vasconcelos, 2003; Monath, 2008). The transmission of YFV, like other arboviruses, is a cycle of infections involving invertebrate hosts (mosquitoes) and a mammal species (non-human primates-NHP and humans). In South America, two transmission cycles have been recorded: the sylvatic cycle, which takes place essentially in rainforests and trees canopy, where the amplifying hosts are NHP, and the vectors are mainly mosquitoes of the genera *Haemagogus* and *Sabethes*; and the urban cycle, where the virus is transmitted between humans by the domestic mosquito vector *Aedes aegypti* (Dégallier et al., 1992). *Ae. aegypti*-vectored cases have not been recorded in Brazil since 1942 (Possas et al., 2018).

In Brazil, as in Europe, North America, and Africa, yellow fever caused historical epidemics between the 18th and 20th centuries (Douam and Ploss, 2018). After the development of the attenuated virus vaccine and the efforts to eradicate the urban mosquito vector *Ae. aegypti*, epidemics of yellow fever became sparse (Dégallier et al., 1992; Staples and Monath, 2008). Until the turn of the 21st century, the Brazilian endemic area covered the entire northern and central-western regions, with a transition zone comprising areas in bordering states of the northeast, especially Maranhão and Bahia, and southeast regions, particularly Minas Gerais e São Paulo, and extending to the southern states. From northeast to southern Brazil, the Atlantic coastal area was considered free of yellow fever for several decades (Fundação Nacional de Saúde [FUNASA], 2003). Nevertheless, since early 2000 an increasing viral dissemination toward Brazil’s southern and southeastern regions occurred. Notably, there were enzootic waves, one reaching Minas Gerais (MG, southeast) in 2000 and 2001 (de Filippis et al., 2002) and Rio Grande do Sul (RS, south) in 2003 (Vasconcelos et al., 2003), causing several human cases in MG, and epizooties in RS, and another crossing the western portions of the states of São Paulo (SP, southeast), Paraná (PR, south) and RS, from 2007 to 2010 (de Souza et al., 2010).

A YFV epizootic wave started in 2014 in the Amazon (north) and has been almost continuously spreading southward after successively crossing the states of Goiás (GO, central west) and MG (Brasil, 2019c,2021b). Between 2016 and 2019, one of the most significant events of the YFV history in Brazil took place: the viral dispersal reached the Atlantic coast, where the virus was undetected for decades, and the vaccination coverage was insignificant, causing the most extensive outbreak since the vaccination campaigns that began in 1937 (Franco, 1969). There were 2,523 epizootics, and 2,155 confirmed human cases (case fatality rate of 34.4%) from July 2016 to October 2018 in the states of Minas Gerais, São Paulo, Espírito Santo (ES), and Rio de Janeiro (RJ), all four states located in the southeastern region (Brasil, 2017, 2018). In this occasion, the Brazilian Health Ministry recommended the vaccination of the population in states without vaccine coverage in the Southeast, in the South and some localities in the Northeast until 2019 (Mendes, 2018) and extended the program of vaccination in other localities in the Northeast in 2020 (Monteiro, 2019), this way, the entire country comprises a routine of immunization against YF. In 2018, 8.1 million fractionated doses were distributed to immunize 40.9 million people in SP, RJ and Bahia (Northeast) (Mendes, 2018).

After the winter of 2018, a sharp reduction of epizootic and human cases was observed in the southeast. However, the transmission of YFV has been reported in areas affected by the end of the outbreak, particularly in the states of Paraná and Santa Catarina (south). In these southern states, most cases have been reported from mid-2019 to January 2020 (Brasil, 2019a,b, 2020a). Seasonal monitoring of YFV indicates that YFV circulation persisted in these states until July 2020, with 390 epizootics and 19 human cases. Reports of YFV infections in humans and NHP have been made until May 2021 in RS, the southernmost Brazilian state (Brasil, 2021a). Epidemiological surveillance data advise that after 6 years of YFV spread outside the Amazon region (endemic area), health services should intensify the efforts to detect early and timely the active circulation of the virus in the region. The data suggest that the YFV might continue spreading south-westward and reach other non-endemic areas in South America where yellow fever vaccination is not recommended (Brasil, 2020b).

Since 2004, the spread of yellow fever in Brazil causing outbreaks has been associated with a modern YFV strain belonging to the South America I genotype subclade 1E (de Souza et al., 2010). It was estimated that the last 25 years (the 1990s–2010s) corresponds to a spanning period of the modern sub-clade 1E in Brazil (Mir et al., 2017). Although the YFV circulating since 2016 belongs to the sub-clade 1E, sequences of samples collected from different hosts and vectors during the last outbreak displayed a molecular signature of nine amino acid substitutions (Bonaldo et al., 2017). In this study, we selected four isolates of the sub-clade 1E, two carrying the molecular signature (here referred to as YFV 2016–2019 group), and two without the amino acid substitutions (here referred to as YFV 2000–2010 group), each bearing other distinct genomic variations.

We evaluated and compared the infectivity dynamics of these four YFV strains in cell culture and the neurovirulence in BALB/c mice in order to establish if there were any differences in viral replicative fitness between the YFV 2000–2010 and YFV 2016–2019 groups.

## Materials and Methods

### Viral Isolates

All four viral isolates used in this study originated from blood or liver samples collected from dying or dead howler monkeys, genus *Alouatta* spp., in different states of Brazil. YFV PR4408 was isolated from the serum of an NHP in RS, southern region, in February 2008. YFV GO05 was isolated from an NHP liver sample collected in GO in April 2017 in the central-western region of Brazil (Delatorre et al., 2019). YFV ES-504 was isolated from an NHP found dead in ES in February 2017 (Bonaldo et al., 2017), and RJ 155 was collected in January 2019 in RJ (Abreu et al., 2019), both located in southeastern Brazil. The genomic sequences of these isolates were previously deposited in GenBank under the accession numbers KY861728 (PR4408), MK333803 (GO05), KY885000 (ES-504), and MK533792 (RJ 155). Detailed information about the isolates is described in Supplementary Figure 1. Collection and management of monkey samples were conducted in accordance with the Brazilian environmental authorities (SISBIO-MMA licenses 54707-6 and 52472-2, and INEA licenses 012/2016, 019/2018) and Committee on the Ethics of Animal Experimentation of Oswaldo Cruz Institute (CEUA-IOC; Permit: L037/2016).

### Cell Cultures

Vero cells (ATCC-CCL81) were maintained in Earle’s 199 medium (Gibco, Thermo Fisher Scientific, United States) supplemented with 5% fetal bovine serum (FBS; Gibco, Thermo Fisher Scientific, United States), 0.25% sodium bicarbonate (Sigma-Aldrich, United States), and 40 mg/mL gentamicin (Gibco, Thermo Fisher Scientific, United States). HepG2 cells (JCRB1054, JCRB Cell Bank) were cultivated in DMEM medium (Gibco, Thermo Fisher Scientific, United States) supplemented with 10% FBS, 1% (MEM) non-essential amino acids (NEAA; Gibco, Thermo Fisher Scientific, United States), 1 mM sodium pyruvate (Gibco, Thermo Fisher Scientific, United States) and 100 U/mL penicillin-streptomycin (Gibco, Thermo Fisher Scientific, United States). Both cell lines were incubated at 37°C with a wet atmosphere and 5% CO_2_. C6/36 cells, provided by Dr. Anna-Bella Failloux (Institut Pasteur, Paris), were grown in Leibovitz’s L-15 medium (Gibco, Thermo Fisher Scientific, United States), supplemented with 5% FBS, 10% tryptose broth, and 40 mg/mL gentamicin. Aag2 cells, provided by Dr. Rafaela Vieira Bruno (Instituto Oswaldo Cruz, Fiocruz/RJ), were seeded in Schneider’s Insect medium (Gibco, Thermo Fisher Scientific, United States), supplemented with 10% FBS and 100 U/mL penicillin-streptomycin. C6/36 and Aag2 cell lines were maintained in an incubator at 28°C.

### Viral Isolation and Obtention of Viral Stocks

C6/36 and Vero cells were seeded at 80,000 and 40,000 cells/cm^2^, respectively, in two T-12.5 flasks. Volumes of 50 and 150 μL of NHP sera or filtered suspension of liver homogenate in Leibovitz’s L-15 medium were centrifuged at 14,000 *g* for 2 min, then transferred to a new 1.5 mL centrifuge tube and diluted in appropriate supplemented medium (with 20% FBS instead of 5%) to a final volume of 0.5 mL. The cell culture medium was removed, and the viral samples were added to the cell monolayers. After 5 min of incubation, cells usually lose from the flask bottom. Hence, fresh cells were added to the flask with more than 5 mL of 20% FBS supplemented medium. Clots were removed with a serological pipette when formed during infection. Vero cells were monitored daily for cytopathic effect, indicating viral infection. For C6/36 cells, 5–8 days post-infection (dpi) infection, aliquots of cell supernatant were collected for RNA extraction followed by RT-PCR for viral detection, confirming the isolation. For both cell lines, RNA was extracted with QIAmp Viral RNA Mini Kit (Qiagen, Germany) according to the manufacturer’s instructions, and RT-PCR was performed as described elsewhere (Bonaldo et al., 2017). The cells supernatants were collected, centrifuged at 400 *g* for 5 min at 4°C, filtered (0.22 μm; Millipore), and stored at −80°C. ES-504, PR4408, and GO05 isolates were isolated in C6/36 cells, and RJ 155 was isolated in Vero cells.

These cell supernatants were then used to obtain the viral stocks, infecting C6/36 cells seeded at 80,000 cells/cm^2^ with supplemented Leibovitz’s L-15 medium in two T-75 flasks. In each flask, after medium removal, 2.5 mL of viral sample were directly added to the cells and incubated at 28°C for 1 h, with agitation every 15 min. After incubation, 40 mL of fresh medium were added to the cells. Seven days after infection, cell supernatants were collected, centrifuged at 400 *g* for 5 min at 4°C, filtered and stored at −80°C. Viral stocks were titrated by plaque assay. The entire genomes were sequenced (Gomez et al., 2018) and compared to the previously determined genome sequences of the four YFV isolates to check the genomic identity (Bonaldo et al., 2017; Abreu et al., 2019; Delatorre et al., 2019). Viral stocks of ES-504 were obtained after two passages and GO05, after one passage in C6/36 cells. RJ 155 was passaged once in Vero cells followed by three additional passages in C6/36, and PR4408 was passaged three times in C6/36, once in Vero, followed by two additional passages in C6/36.

### Viral Titration by Plaque Assay

Viral suspensions were serial diluted 1:10 six times, and 100 μL of each dilution were added to Vero cells monolayer, previously seeded at 50,000 cells/cm^2^ in a 24-well plate (24 h before inoculation). After 1 h incubation at 37°C and 5% CO_2_, the viral inocula were aspirated and cells were covered with 1 mL of supplemented Earle’s 199 medium containing 2.4% carboxymethylcellulose (CMC). Cells were then incubated for 10 days at 37°C, 5% CO_2_, followed by fixation with 1 mL 10.0% formaldehyde overnight. The monolayer was washed with water to remove residual formaldehyde and cell culture medium and stained with 0.4% crystal violet.

### Plaque Phenotype Assay

Vero cells were seeded in 6-well plates at a density of 40,000 cells/cm^2^, approximately 24 h before infection. For each virus, three inocula were prepared with supplemented Earle’s 199 medium containing 10, 20, and 40 PFU in 0.2 mL of viral suspension. Culture supernatant was removed entirely, and 200 μL of viral inoculum were added to the cell monolayer. After 1 h incubation at 37°C and 5% CO_2_ with gentle agitation every 10 min, the inocula were discarded, and the cells were overlaid with 3 mL of supplemented Earle’s 199 medium containing 0.5% agarose (Invitrogen, Thermo Fisher Scientific, United States). Cells then returned to the incubator at 37°C and 5% CO_2_ for 7 days, after which cells were fixed with 5 mL 10.0% formaldehyde overnight and stained with 0.4% violet crystal. Images of the plates were acquired and analyzed using ImageJ software to measure plaque areas. The results were plotted into graphs and statistically analyzed using GraphPad Prism software 8. Statistical tests employed were Kruskal–Wallis with Dunn’s multiple comparison test.

### Cell Viability Assay

Vero cells were cultivated in a 96-well microplate at a density of 20,000 cells/well 24 h before infection. The viral inoculum was prepared in Earle’s 199 medium with 5% bicarbonate and no addition of SFB. Cell supernatant was discarded, and 50 μL of viral suspension were added to the cell monolayer, at MOI 0.1, followed by 2 h incubation at 37°C, 5% CO_2_. Then, the viral suspensions were removed, and 90 μL of supplemented Earle’s 199 medium were added to each well. After 24 h of incubation at 37°C and 5% CO_2_, 10 μL of PrestoBlue Reagent (Invitrogen, Thermo Fisher Scientific, United States) were added to each well and incubated for 15 min at 37°C. Absorbance measures were acquired with SoftMax Pro 6.5 software using VersaMax Tunable Microplate Reader (Molecular Devices), at wavelength 570 nm normalized at 600 nm. Data were analyzed in GraphPad Prism software 8 with one-way ANOVA followed by Tukey’s multiple comparisons test.

### Viral Infection in Cells Treated With Type I Interferon

Vero cells were grown in 24-well plates at a density of 50,000 cells/cm^2^. The next day, cells were treated with interferon (IFN) alpha (α; PBL Assay Science, United States) and beta (β; R&D Systems, United States) at concentrations of 10, 50, 100, and 1,000 UI/mL for 6 h before infection. Cell supernatant was removed, and 100 μL of viral suspensions were added at MOI of 0.5. After 1 h incubation with gentle agitation every 15 min, viral inocula were discarded, and cells were overlaid with 0.3 mL of supplemented Earle’s 199 medium, containing the four different IFN concentrations. After 48 h incubation at 37°C and 5% CO_2_, the supernatants were collected for viral titration by plaque assay. The log_10_ of viral titers under treatment with IFN-α and IFN-β were normalized with the values obtained from non-treated infected cells. This experiment was performed in three independent assays for statistical relevance. Data were analyzed in GraphPad Prism 8 software. IC_50_ values were calculated from the non-linear regression function provided by the software [(Inhibitor) vs. normalized response – Variable slope]. IC_50_ values of each replicate were analyzed by one-way ANOVA with Bonferroni’s multiple comparison test.

### Viral Growth in Mosquito and Mammal Cells

Cells were seeded at different densities 24 h before infection. Insect cells, Aag2 and C6/36, were seeded at 80,000 cells/cm^2^, Vero cells at 40,000 cells/cm^2^, and HepG2 at 60,000 cells/cm^2^ in T-25 culture flasks. Viral stocks were diluted to infect cell monolayers with an MOI of 0.02. Growth culture media were discarded, and 0.5 mL viral samples were added to the cells. After 1 h incubation with gentle agitation every 15 min, viral inocula were completely removed, and 12 mL of appropriate cell culture medium were added. Cell cultures were observed daily, and aliquots of supernatant were collected every 24 h until 5 days to determine viral titers at each day post-infection. Viral titration was carried out by plaque assay, and the growth curves were analyzed using GraphPad Prism 8 software. Statistical tests employed were one-way ANOVA with Bonferroni’s multiple comparison test.

### Neurovirulence in BALB/c Mice

BALB/c mice were obtained from CEMIB (Centro Multidisciplinar para Investigação Biológica na Área da Ciência em Animais de Laboratório), of the State University of Campinas, São Paulo (UNICAMP). This experimentation was carried out in strict accordance with the Guide of the National Council for Control of Animal Experimentation (CONCEA). The protocols employed were approved by the Committee on the Ethics of Animal Experimentation of Oswaldo Cruz Institute (CEUA-IOC; Permit: L-034/2019). Groups of five young adult BALB/c mice (6 weeks old) were inoculated intracerebrally with a dose of 10^3^ PFU in a final volume of 30 μL of each isolate virus or the vaccine YFV 17DD (Instituto de Tecnologia em Imunobiológicos, Bio-Manguinhos) as a positive control. Mock-infected mice were inoculated with a diluent medium in which the viral inocula were prepared (Earle’s 199 medium supplemented with 25 mM HEPES and 0.025% sodium bicarbonate). The inoculation occurred under anesthesia with a Ketamine/Xylazine cocktail at a dose of 100 and 10 mg/kg, respectively, administered intraperitoneally. The animals were monitored daily for 21 days with an evaluation of clinical signs of disease and weight measurement. Clinical scores were established to determine the humane endpoint for euthanasia of mice (Supplementary Table 2). Evaluated clinical signs included the percentage of body weight loss, ruffled fur, hunched posture, low mobility, paralysis of posterior members, aggressiveness, and respiratory disorders. Euthanasia was performed with intraperitoneal administration of a three times more significant dose of Ketamine/Xylazine cocktail, followed by cervical dislocation. This experimentation was reproduced in triplicate, totalizing 15 animals infected with each viral sample. Average survival time (AST), percentage of mortality, clinical scores, and body weight loss were calculated and analyzed in GraphPad Prism 8 software. Statistical analysis of Kaplan–Meier survival curves was performed by log-rank test (Mantel–Cox).

### Sequence Analysis

Yellow fever virus isolates sequences were aligned using ClustalW algorithm available in MEGA Software v. 7.0.26. The variable sites were highlighted in MEGA Software and exported in Excel format for better representation. The amino-acid and nucleotide alignments were analyzed in BioEdit Sequence Alignment Editor 7.2.6.1 to calculate their respective identity matrix.

### Phylogenetic Analysis

Yellow fever virus complete genome sequences were retrieved from the GenBank database and aligned using AliView v.1.27 (Larsson, 2014). Phylogenetic reconstruction was conducted using IQ-TREE software multicore version 2.1.2 (Minh et al., 2020); it inferred the Maximum Likelihood tree from the input alignment with the best fit model automatically selected by ModelFinder (Kalyaanamoorthy et al., 2017). The ML analysis was carried out with 1,000 bootstrap replicates. The obtained tree was edited using FigTree v.1.4.4.

## Results

### Genome Analyses of the Yellow Fever Virus Isolates

The four YFV investigated in this study were isolated from infected howler monkeys in the states of RS in 2008, GO and ES in 2017, and RJ 2019. Here, the isolates were grouped according to the presence (YFV 2016–2019) or absence (YFV 2000–2010) of the molecular signature described in 2017 (Bonaldo et al., 2017).

Although GO05 was identified after 2010, this isolate does not carry the molecular signature. Thus, both PR4408 and GO05 (Delatorre et al., 2019) viral isolates belong to the YFV 2000–2010 group. They were sampled in RS in 2008 and GO in 2017, respectively. The isolates ES-504 (Bonaldo et al., 2017) and RJ 155 (Abreu et al., 2019) belong to YFV 2016–2019. They were obtained from samples collected in ES in 2017 and RJ in 2019, respectively (Supplementary Figure 1).

Before we deepen into the cellular and mouse infection characterization, we established the genomic features inherent to each isolate. Initially, to determine the genetic differences between the four isolates, we retrieved their genomic sequences from the GenBank database, aligned them, and calculated nucleotide and amino acid identities. As shown in Table 1, the identity matrix revealed that the four isolates are very similar and shared 99.4–99.8% of amino acid identity, while nucleotide analysis showed lower scores (from 98.2 to 99.8%). This difference is due to the higher rate of synonymous (84.7%) than non-synonymous (10.0%) genomic variations, as detailed in Supplementary Table 1. The two YFV 2016–2019 isolates, ES-504 and RJ 155, display high identity scores between them, both at the amino acid (99.8%) and nucleotide levels (99.8%). The same trend was observed between PR4408 and GO05 isolates exhibiting 99.7 and 98.6% of polyprotein and genome sequence identities, respectively.

**TABLE 1:**
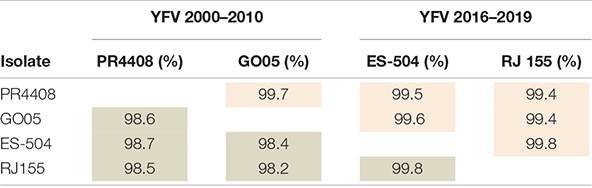
Identity matrix of the polyprotein and the genome of YFV isolates.*

*Values highlighted in light red represent amino acid identity rates and those in gray, the identity in terms of the genome nucleotide sequence.

The alignment of the four isolates’ polyproteins firstly revealed the set of nine amino acid polymorphisms that distinguishes the southeast Brazilian outbreak 2016–2019 YFV lineages (Table 2 and Supplementary Figure 2). The molecular alterations mapped at the C protein (V108I; C residue 108), at NS3pro (E1572D; R1605K; NS3 residues 88 and 121, respectively), at NS5 in MTase domain (K2607R; V2644I; G2679S; NS5 residues 101, 138 and 173, respectively) and NS5 in the RdRp domain (N/D2803S; V3149A; N3215S; NS5 residues 297, 643 and 709, respectively).

**TABLE 2:**
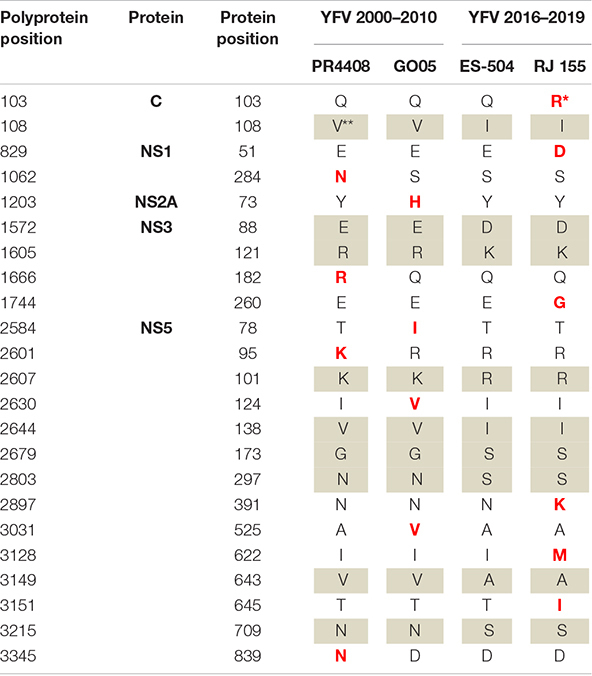
Amino acid variations observed in the polyprotein of the YFV isolates.

*Amino acids in bold red represent the unique amino acid occurrence in a certain isolate YFV.

**Highlighted in gray, the set of nine amino acid variations that characterizes the YFV 2000–2010 and YFV 2016–2019 samples.

Each isolate displays specific amino acid variations in comparison to each other. Most amino acid differences are localized at the NS proteins (21 out of 23), with the majority mapping at NS5 protein (14 out of 21). More specifically, the PR4408 and GO05 carry four aminoacidic modifications and the NS proteins, while RJ 155 presents six, with one in the capsid (C) structural protein. The isolate ES-504 carries no unique modification. It is noteworthy that the isolate RJ 155 holds three unique residues clustered in the RNA-dependent RNA-polymerase (RdRp) domain of the NS5 protein (Table 2).

Further, the isolates’ UTR were aligned and revealed that the YFV 2000–2010 exhibit some variations, especially in the 3′UTR region, compared to the YFV 2016–2019 (Supplementary Table 1). Remarkably, the GO05 isolate cumulates more exclusive changes in the UTRs, one in the 5′UTR and 9 others in the 3′UTR.

Furthermore, phylogenetic analysis showed that ES-504 and RJ 155 cluster inside the same clade, while GO05 and PR4408 are in two different branches with high-supported nodes (>90% of bootstrap value) (Supplementary Figure 3). The phylogenetic tree demonstrates that YFV PR4408 did not cluster with the other three isolates, but in a separate branch with a well-supported bootstrap value of 99%. The GO05 isolate is not clustered with the other two YFV (ES-504 and RJ 155) in 92% of the iterations. Together with the identity matrix analyses, these results support that ES-504 and RJ 155 were more genetically related. In contrast, the isolate PR4408 was the most genetically distinct genome of all the four YFV studied.

### Viral Growth Kinetics in Different Cell Lines

**FIGURE 1:**
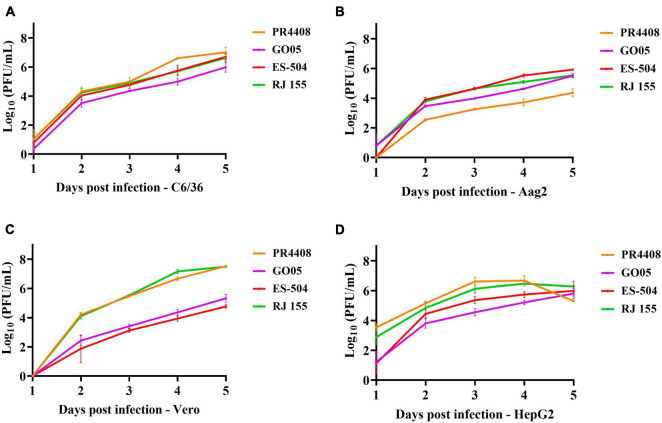
Viral isolates replication kinetics in different cell lines. The replication profiles obtained from mosquito **(A)** C6/36 and **(B)** Aag2 cell lines, and from mammal Vero **(C)** and HepG2 **(D)** cell lines. Cells were infected with the YFV isolates at MOI 0.02 and supernatants were used to determine viral titers each day post infection for 5 days. Statistical analyses applied were Ordinary one-way ANOVA with Bonferroni’s multiple comparison test (Supplementary Figure 3) using GraphPad Prism 8.

The subsequent experimental procedures were performed with the viral stocks obtained from infection in cells with each isolate. The complete genomes after cell passage were sequenced to verify genomic identity with the data deposited in GenBank Database (data not shown). To evaluate the potential effect of the detected polymorphisms in cell infectivity, we infected two mammal (Vero and HepG2) and two mosquito (C6/36 and Aag2) cell lines with the four YFV isolates at MOI of 0.02. In the *Aedes albopictus* mosquito cells, C6/36, the proliferation rates are highly similar among isolates (Figure 1A). The GO05 virus presented the lowest titers along the 5 dpi, with statistical significance at 4 and 5 dpi (Supplementary Figure 4). The C6/36 growth curves displayed an ascending profile reaching no viral replication peak. On the other hand, the curves in *Ae. aegypti* Aag2 cells reached a viral growth peak at 4 dpi. In Aag2 cells, PR4408 and GO05 replicated less than RJ 155 and ES-504 (Figure 1B). In general, the viral yields at 4 and 5 dpi obtained in both mosquito cells were similar. The exception was PR4408 that exhibited a reduction of 450 (4 dpi) and 780 (5 dpi) times in proliferation rates in Aag2 cells (Supplementary Figure 5). These results point to better infectivity of the YFV 2016–2019 than the YFV 2000–2010 isolates in mosquito cells.

In mammal Vero and HepG2 cells, we could not establish a pattern associated with the group of isolates. YFV GO05 and ES-504 displayed lower titers than RJ 155 and PR4408 (Figures 1C,D). Replication curves of YFV isolates in Vero and HepG2 reached viral growth peaks at 3 and 4 dpi, respectively. The most notable differences occurred in the viral proliferation in Vero cells (Figure 1C): RJ 155 and PR4408 isolates yielded significantly higher viral titers than GO05 and ES-504. While RJ 155 and PR4408 exhibited nearly identical curves in both mammalian cells, GO05 and ES-504 were slightly different in HepG2 cells. The statistics of the viral replication kinetics are detailed in Supplementary Figures 4, 5.

### Plaque Phenotype

**FIGURE 2:**
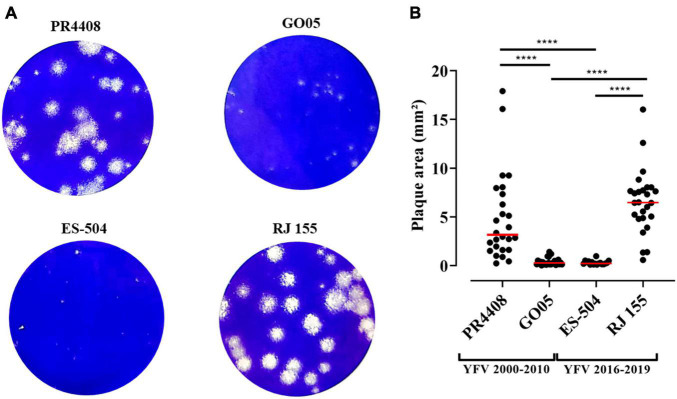
Viral plaque phenotype determination of the YFV 2000–2010 and YFV 2016–2019 isolates. **(A)** Infected Vero cells stained with crystal violet. **(B)** Scatter plot graphs indicating the individual values for plaque areas. Red bars represent the mean of the data sample. The plaque areas were calculated using ImageJ software. Data were analyzed in GraphPad Prism 8 software. The plaque means were statistically analyzed by Kruskal–Wallis with Dunn’s multiple comparisons test. ****Represents P ≤ 0.0001.

To further characterize the YFV isolates, they were defined by their plaque phenotype in Vero cells. As observed in Figure 2, RJ 155 and PR4408 exhibited larger and more diverse plaque sizes (average plaque areas: 6.54 ± 3.28 and 4.90 ± 4.47 mm^2^, respectively). The isolates ES-504 and GO05 had more homogenous plaques in size and smaller areas (mean of areas: 0.31 ± 0.21 and 0.41 ± 0.38 mm^2^, respectively). Although the mean plaque area of RJ 155 was larger than the mean of PR4408 plaques, there was no significant difference between them (*P* = 0.1812). Likewise, ES-504 and GO05 presented a slight difference in mean that was not statistically significant (*P* = 0.9995). Comparison between isolates belonging to the same group, YFV 2016–2019 (ES-504 and RJ 155) and YFV 2000–2010 (GO05 and PR4408), showed significantly different plaque phenotypes (*P* < 0.0001).

### Viral Replication in Vero Cells in the Presence of Type I Interferon

Additionally, we submitted Vero cells to treatment with interferon-alpha and beta (IFN-α and IFN-β) in four different concentrations: 10, 50, 100, and 1,000 UI/mL. Cells were treated before and after viral adsorption with each YFV isolates at MOI 0.5. Viral concentrations in cell supernatants after 48 h of incubation were calculated and analyzed. The IC_50_ values for infection under IFN-α and IFN-β treatment with each YFV were calculated through a non-linear regression curve fitting.

**FIGURE 3:**
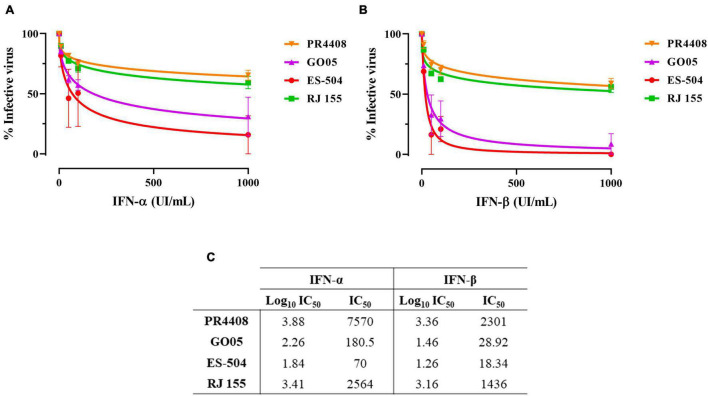
Infectivity in Vero cells treated with IFN-I. Non-linear regression of viable YFV after 48 h of infection in Vero cells under treatment with **(A)** IFN-α and **(B)** IFN-β. **(C)** IC_50_ values obtained for all YFV isolates after treatment with IFN-α and IFN-β. Data was analyzed with GraphPad Prism 8 software. The curves were fitted by non-linear regression of normalized data with variable slope.

The results obtained were similar for both IFN-α and IFN-β, although all four YFV isolates tended to be less sensitive to IFN-α than to IFN-β (Figures 3A,B, respectively). YFV PR4408 and RJ 155 displayed higher viral titers despite the treatment with IFN, with IC_50_ values of 7,570 and 2,564 UI/mL for IFN-α, and 2,301 and 1,436 UI/mL for IFN-β, respectively. Unlike YFV GO05 and ES-504, which were more strongly inhibited by IFN treatment, presenting IC_50_ of 180.5 and 70 UI/mL for IFN-α, and 28.92 18.34 UI/mL for IFN-β, respectively (Figure 3C).

The IC_50_ values were also submitted to the statistical one-way ANOVA test with Bonferroni’s multiple comparison test to evaluate if the differences are statistically relevant (Supplementary Figure 6). The treatment with IFN-α influenced less in the infectivity of the YFV. The only statistical difference was observed between IC_50_ of PR4408 and ES-504 (*P* = 0.043). On the other hand, the IC_50_ values obtained after treatment with IFN-β were significantly different between PR4408 and GO05 (*P* = 0.003), PR4408 and ES-504 (*P* = 0.001), RJ 155 and GO05 (*P* = 0.006), and RJ 155 and ES-504 (*P* = 0.002). In summary, ES-504 and GO05 did not display differences in IC_50_ values, as well as RJ 155 and PR4408. Altogether, YFV PR4408 and RJ 155 exhibited similar IC_50_ values, at least 15 times higher than the IC_50_ of GO05 and ES-504.

### Virus-Induced Cytotoxicity

**FIGURE 4:**
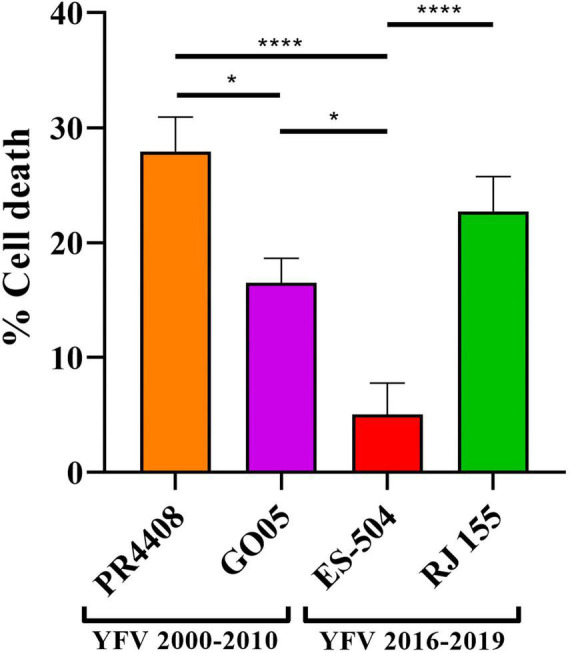
Virus-induced cytotoxicity in Vero cells at 24 h post infection at MOI 0.1. Statistical analyses were performed in GraphPad Prism 8, by Ordinary one-way ANOVA with Tukey’s multiple comparisons test: * represents *P* ≤ 0.05 and **** represents *P* ≤ 0.0001.

We also compared the YFV isolates in terms of their ability to promote cytotoxicity in Vero cells. After 24 h of infection with the different YFV isolates at MOI 0.1, the cell viability was determined in infected Vero cells (Figure 4). The isolates RJ 155 and PR4408 induced the highest percentages of cell death (22.72 and 27.93%, respectively). GO05 displayed an intermediary cytotoxic effect with 16.50% of cell death. YFV ES-504 reached the lowest cytotoxicity (5.04%). As observed in mammal cell infection assays, we can establish a pattern associated neither with the YFV 2000–2010 or YFV 2016–2019 groups. Statistical analyses showed significant differences between the isolates, except RJ 155 and GO05, and RJ 155 and PR4408 (Figure 4).

### Neurovirulence in BALB/c Mice

**FIGURE 5:**
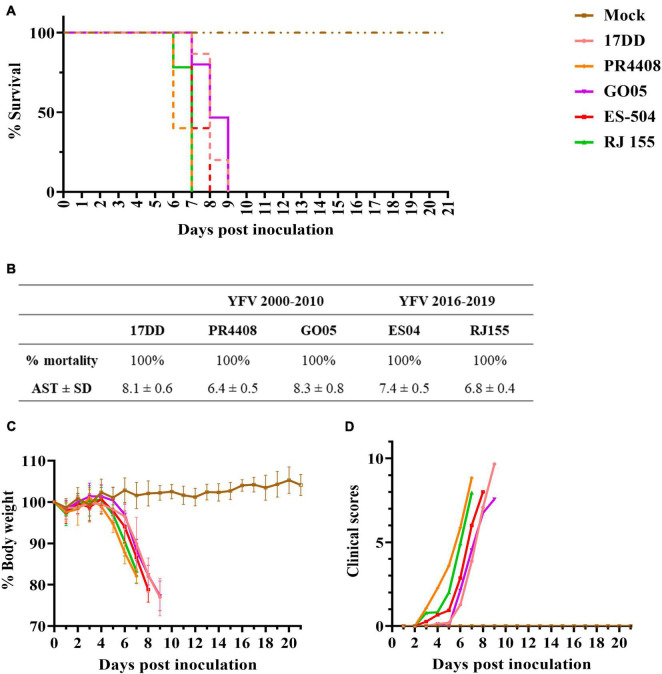
Neurovirulence of YFV isolates in BALB/c mice. Six-week-old mice were inoculated intracerebrally with 10^3^ PFU of the isolates or diluent medium (Mock). Mice were followed up daily for 21 days. The average survival times were deduced from Kaplan–Meier survival curves **(A)** and summarized in table **(B)**. Morbidity was measured by the percentage of body weight loss **(C)** and clinical scores **(D)**.

The final comparative study between YFV 2000–2010 and 2016–2019 isolates was a neurovirulence test in the mouse model. Intracerebrally inoculated mice were monitored daily for bodyweight loss and clinical signs of disease, such as ruffled fur, hunched posture, difficult breathing, repetitive movements, and lethargy (clinical scores are listed in Supplementary Table 2). All YFV isolates led to a 100% mortality (Figures 5A,B) but presenting different AST. Mice infected with YFV isolates RJ 155 and PR4408 had similar AST (6.8 ± 0.4 and 6.4 ± 0.5 days, respectively). In comparison, ES-504 generated an intermediate AST value of 7.4 ± 0.5 days. The isolate GO05 induced an AST of 8.3 ± 0.8 days, comparable to the AST of mice infected with the vaccine YFV 17DD (AST of 8.1 ± 0.6 days). The survival curves were statistically analyzed using the Log-rank (Mantel–Cox) test to determine if the differences observed were significant. Between YFV of the same group, ES-504 and RJ 155 were significantly different with *P* = 0.006 and GO05 and PR4408 with *P* < 0.0001. The least significant differences were observed between RJ 155 and PR4408 (*P* = 0.0184) and ES-504 and GO05 (*P* = 0.0016). Compared with the control group YFV 17DD, only GO05 exhibits no statistical differences (*P* = 0.2952).

The four viruses induced the same loss of body weight and disease outcome in all animals (Figures 5C,D). As soon as 3 dpi, mice infected with isolates PR4408 and RJ 155 showed signs of illness, progressing to a constantly increasing body weight loss after day 5. Animals infected with these viruses reached the critical humane endpoint around the sixth day. ES-504 infected mice start becoming affected by YFV after 4 dpi, with an AST of 7 days. The isolate GO05 proved to be less neurovirulent, with a disease progression similar to the vaccine YFV 17DD concerning weight loss, clinical scores, and AST (Figure 5).

## Discussion

Yellow fever virus has a specific dispersal: it is established that in regions where YFV is maintained mainly in sylvatic cycles, in West Africa and North/South America, the evolution follows an *in situ* pattern (Carrington and Auguste, 2013; Mir et al., 2017). Particularly in South America, the most dispersed genotype, SA I, is marked by the Brazilian diversity and is subdivided into clades that are temporally and geographically clustered (Vasconcelos et al., 2004). Phylogenetic studies demonstrate that this subdivision is due to a lineage replacement dynamic where emerging sub-lineages cause outbreaks and ancestor lineages cease to circulate or disappear (Zanotto et al., 1996). Since 1999, through the emergence of YFV modern-lineage, yellow fever has come back in regions of Brazil where it had been silent for several decades (de Filippis et al., 2002). Modern sub-lineage 1E has caused Brazilian outbreaks since 2004, prevailing in the country, and successfully replaced modern sub-lineage 1D.

In this study, we performed the biological characterization of four sub-lineage 1E isolates from Brazil. All the viral samples were isolated from YFV infected howler monkeys (*Alouatta* spp.), highly susceptible hosts for YFV (de Azevedo Fernandes et al., 2021), whose infection generally resulted in animal death. Therefore, although obtained at different times, all four isolates are YFV virulent to primates circulating in forested environments. A molecular signature is associated with the YFV 2016–2019 outbreak strains, consisting of nine non-synonymous substitutions, present in most sub-lineage 1E sequences resolved since 2016 (Bonaldo et al., 2017; Gomez et al., 2018). Therefore, we considered this diversity and selected isolates carrying or not these molecular markers. The four selected YFV isolates emerged in a time-spanning period from 2008 to 2019 and in different Brazilian geographic regions: south (PR4408), central west (GO05), and southeast (ES-504 and RJ 155).

In the YFV 2000–2010 group, isolate PR4408 is related to a large outbreak in 2008 in RS, the southernmost state in Brazil bordering Uruguay and Argentina, and GO05, circulated in GO in 2017 (central west). Both YFV are more phylogenetically related to the Venezuelan YFV genomic sequences, displaying a similar amino acid pattern to previous YFV sub-clade 1E strains sampled in Brazil and Venezuela between 2000 and 2010 (Delatorre et al., 2019).

The spread of YFV during the outbreak in southeastern Brazil occurred along two main routes from the central west toward the southeastern state of MG (Gomez et al., 2018). We chose the second group of isolates (YFV 2016–2019) related to one of the routes belonging to the YFV MG/ES/RJ sub-lineage (Delatorre et al., 2019). YFV ES-504 was isolated at the beginning of the southeastern outbreak in February 2017 in ES (Bonaldo et al., 2017; Gomez et al., 2018). The isolate RJ 155 obtained in early 2019 corresponds to the only and last record of YFV circulation in RJ. Indeed, it consists of the first sign of virus re-emergence in the state since July 2018 (Abreu et al., 2019). More specifically, this sample was detected in an area where the outbreak had occurred two transmission seasons before, indicating that the virus transmission can be resilient in the Atlantic Forest zone, independently of new introductions (Abreu et al., 2019).

These four isolates exhibit a high degree of genetic similarity. Most amino acidic variations are localized in the NS proteins (21 out of 23), mainly in NS5 (14 out of 21). In this work, we intended to investigate if these polymorphisms may impact viral fitness leading to phenotypic differences in viral virulence, consisting in a first approach to establish the genotypical basis of this feature. To understand the genetic dynamic of YFV, we must consider that RNA viruses evolve at higher rates than DNA viruses (Drake et al., 1998). However, RNA viruses transmitted by arthropod vectors to mammals, like flaviviruses, seem to display reduced rates of non-synonymous mutations. The viral propagation in multiple hosts implies restrictions in fixing changes in viral proteins, as these might cause a reduction in fitness in one of the hosts and be deleterious. There is a positive selection of the most mutations occurring in flaviviruses that alternately infect insect and mammalian hosts (Woelk and Holmes, 2002). Genetic changes in these viruses should not considerably disturb the viral replication in the other. Thus, amino acid alterations might modulate the infection in one host without disturbing the viral replication in the other.

To evaluate the differences among YFV isolates abilities to infect mosquito and mammal hosts, we initially utilized mosquito cells to assess the influence of genetic variability on infection. Comparing the infection of Aag2 mosquito cells by the isolates, we noticed a possible influence of the YFV 2016–2019 molecular signature in infectivity, with lower viral yields achieved by isolates without this polymorphism. The effect is only observed in Aag2 cells with no apparent differences in viral proliferation in C6/36 cells, and it is more evident in the YFV PR4408 proliferation rates. C6/36 cells promote, in general, a robust growth of arthropod-borne viruses because they are defective in the expression of siRNA production, an important antiviral defense against arboviruses in mosquitoes (Brackney et al., 2010). On the other hand, Aag2 cells efficiently mount an antiviral response inducing the RNAi pathway (Brackney et al., 2010). Consequently, the viral proliferation in these cells is more controlled and restrictive. The potential increased fitness of the YFV 2016–2019 molecular signature in mosquito adaptation needs to be tested by determining vector competence in *Aedes*, *Haemagogus*, and *Sabethes* mosquito populations.

In mammal cells, Vero and HepG2, we did not detect any correlation between isolates carrying the YFV 2016–2019 molecular signature and growth kinetics. PR4408 and RJ 155 display higher rates of proliferation than ES-504 and GO05 isolates. These results match the plaque phenotype and cell viability assays, where RJ 155 and PR4408 exhibit larger plaques and more evidently induce cell death. These properties would reflect an increased viral capacity to replicate in mammal cells (Collette et al., 2020). On the other hand, it might also be related to the more effective modulation of the cellular antiviral response, primarily triggered by IFN-I. It is well established that the ability to antagonize the IFN-I response is an essential determinant of virus virulence (Best, 2017). Here we observed that PR4408 and RJ 155 isolates displayed higher viral yields than GO05 and ES-504 isolates in the presence of IFN-I (α and β), as expected.

The virulence of the four viruses was investigated by intracerebral inoculation in mice. The neurovirulence test in mice has been utilized to compare vaccine YFV, wild-type strains, or mutant variants (Barrett and Gould, 1986; Muylaert et al., 1996; Xie et al., 1998; Chambers and Nickells, 2001; Vlaycheva and Chambers, 2002). In general, vaccines and isolates YFV provoke 100% death of inoculated animals. The main parameter utilized in comparison among YFV strains is the AST reached for every virus. Barrett and Gould (1986) have performed neurovirulence tests with different vaccine strains, including 17DD, produced in Brazil, the same seed vaccine strain used as a positive control in the present study. Our data corroborated a more neurovirulent phenotype of RJ 155 and PR4408, exhibiting AST between 6 and 7 days after intracerebral inoculation, compared to ES-504 and GO05 isolates, with AST between 7 and 8 dpi.

Our study did not observe any apparent influence of the YFV 2016–2019 molecular signature on infection rates in the invertebrate hosts. Here, the most infective viruses in mammal cell models or mouse infection were YFV PR4408, not carrying the molecular signature, and RJ 155, with the nine amino acid modifications observed in the YFV 2016–2019 group. The differences in the infective properties observed in this work could be related to the precursor-polyprotein polymorphisms. Except for the YFV ES-504 isolate, the three other YFV strains carry unique amino acid variations that could modulate the distinct virological properties observed in this study. In the case of YFV 2016–2019, the isolates ES-504 and RJ 155 clustered in a monophyletic group showing high genomic similarity, whereas isolates of the 2000–2010 group clustered in two separate branches (Supplementary Figure 3). Between ES-504 and RJ 155, we observed 21 nucleotide variations, six of them causing amino acid changes. The set of nucleotide variations consists of one in 3′UTR and 13 silent changes in the precursor-polyprotein codifying sequence. We can speculate that the silent mutations and the change in 3′UTR might cause an effect in modulating viral infectivity changing RNA secondary structures (Proutski et al., 1997). On the other hand, the six amino acid changes may cause the virological differences observed between the isolates of YFV 2016–019.

Isolates RJ 155 and PR4408 display a similar infection pattern in mammalian cells and mice. While YFV RJ 155 carries six unique amino acid modifications, one in C protein (C 103) and five in NS proteins (NS1 51; NS3 260; NS5 391, 622, and 645), isolate PR4408 does not present any of these six amino acid markers, suggesting that other genetic polymorphisms might be responsible for higher infectivity rates observed in these mammal models. In addition, PR4408 bears four specific amino acid variations inside NS proteins (NS1 284; NS3 182; NS5 95 and 839); these amino acid markers might be related to the infection phenotype, that is, a more restrictive proliferation in mosquito Aag2 cells and higher virulence in the mouse model. On the other hand, the other YFV 2000–2010 virus, the isolate GO05, is considerably more attenuated and carries four specific amino acid alterations inside two NS proteins (NS2A 73; NS5 78, 124, and 525). It is not possible yet to determine the role of the eight amino acid polymorphisms in virulence modulation of the YFV 2000–2010 group. Although YFV PR4408 and GO05 are genetically closer, they are more distant evolutionally than the isolates of the YFV 2016–2019 group (Supplementary Figure 3), reflecting in the phenotypic perspectives where they are too different both *in vitro* and *in vivo*, as shown in our results. Furthermore, it is noteworthy that YFV 2016–2019 were isolated spatially and temporally close, while in YFV 2000–2010 group, PR4408 was isolated geographically distant and 9 years apart from GO05.

We hypothesize that some of these amino acid variations could be involved in the phenotypic differences observed in this study. However, modulation in viral virulence and attenuation can be mono- or multifactorial, and some of these changes could not significantly affect the processes studied here. The results obtained in this study suggest that the differences in viral replicative fitness among the four Brazilian YFV isolates are mainly related to polymorphisms found in NS proteins. Most amino acid polymorphisms are localized in those NS proteins that form the viral replication complex inside the host cell, which have been described as targets for positive selection in flaviviruses (Sironi et al., 2016). It is not possible to predict if those changes are modulating the viral fitness in nature. Further studies will address this point employing reverse genetics to establish which amino-acid variation or variations are responsible for the modulation of infectivity in cells and mice.

The NS2A and NS1 proteins have been described as cofactors in viral replication. Despite evidence that residue changes in NS2A modulated viral replication (Kummerer and Rice, 2002; Marquez-Jurado et al., 2018), its association with the replication complex is still unclear. However, NS2A plays a vital role in viral assembly through association with the complex C-prM-E and viral RNA (Vossmann et al., 2015; Xie et al., 2019). Meanwhile, the NS1 protein is well described as the main factor for ER membrane remodeling and the formation of the replication organelle through its many hydrophobic surfaces (Ci et al., 2020). This protein is multifunctional and is involved in many interactions and processes within the hosts (Rastogi et al., 2016; Watterson et al., 2016), including immune evasion mechanisms (Akey et al., 2014; Shi et al., 2018).

Finally, both NS3 and NS5 proteins perform key enzymatic activities in viral replication. The NS3 N-terminal domain encodes a serine protease, and the C-terminal an RNA helicase also displaying nucleoside triphosphatase (NTPase) and RNA triphosphatase (RTPase) enzymatic activities (Assenberg et al., 2009; Li et al., 2014). NS5 also contains two domains with distinct enzymatic functions. Its N-terminal domain encodes methyl- and guanylyltransferase (MTase and GTase) (Geiss et al., 2009; Issur et al., 2009), and the C-terminal domain performs as an RNA dependent RNA polymerase (RdRp) (Selisko et al., 2018). These two non-structural proteins interact widely with each other and with host proteins (Le Breton et al., 2011). Thus, non-synonymous changes in these NS proteins could interfere with the viral replication and assembly.

At last, we observed amino acid variations only in one of the structural proteins, the capsid protein (C), which also plays a primary role in the viral assembly of flaviviruses (Kuhn et al., 2002). Additionally, an RNA secondary structure present in the C coding region is a critical determinant for flavivirus replication (Clyde and Harris, 2006; Clyde et al., 2008). Furthermore, in YFV, C protein suppresses the antiviral RNA silencing process in mosquitoes by protecting viral dsRNA from processing *via* dicer (Samuel et al., 2016). Consequently, it might be possible that these alterations would play a role in the modulation of viral infectivity.

In summary, we cannot conclude if YFV 2016–2019 molecular signature plays a pivotal role in cell infectivity or neurovirulence. Instead, we can analyze whether the location and date of sample collection are influenced by genome composition. The phylogenetic analysis (Supplementary Figure 3) demonstrates that YFV PR4408 did not cluster with the other three isolates but in a separate branch with a well-supported bootstrap value of 99%. Hence, the differences between PR4408 and GO05 may be related to a different YFV evolutionary timing in Brazil. Meanwhile, the different aspects highlighted in this work between YFV ES-504 and RJ 155 could be directly associated with their genetic features. This study supports the importance of identifying and studying genetic factors along YFV evolution to better understand the potential of amino acid changes in viral fitness.

## Data Availability Statement

The YFV genome sequences are available in the GenBank database under the accession numbers: KY861728 (PR4408), MK333803 (GO05), KY885000 (ES-504), and MK533792 (RJ155).

## Ethics Statement

Collection and management of monkey samples were conducted in accordance with the Brazilian environmental authorities (SISBIO-MMA licenses 54707-6 and 52472-2 and INEA licenses 012/2016 and 019/2018) and Committee on the Ethics of Animal Experimentation of Oswaldo Cruz Institute (CEUAIOC; Permit: L037/2016). The study involving BALB/c mice was reviewed and approved by the Committee on the Ethics of Animal Experimentation of Oswaldo Cruz Institute (CEUA-IOC; Permit: L-034/2019).

## Author Contributions

NF and MB: study conception. FA, MC, LM, PV, and RL-D-O: field collection of NHP blood and liver samples, processed samples, and proceeded to viral isolation. NF, IR, and AS: YFV sequencing. NF and LR: viral studies. DF, IM, MN, and NF: mouse neurovirulence assays. MG: phylogenetic analyses. MB and NF: manuscript preparation. All authors critically read and approved the final version of the manuscript.

## Conflict of Interest

The authors declare that the research was conducted in the absence of any commercial or financial relationships that could be construed as a potential conflict of interest.

## Publisher’s Note

All claims expressed in this article are solely those of the authors and do not necessarily represent those of their affiliated organizations, or those of the publisher, the editors and the reviewers. Any product that may be evaluated in this article, or claim that may be made by its manufacturer, is not guaranteed or endorsed by the publisher.
