# Supplementary material for: Biological Characterization of Yellow Fever Viruses Isolated From Non-human Primates in Brazil With Distinct Genomic Landscapes
Source: Front Microbiol. 2022 Feb 14;13:757084. doi: 10.3389/fmicb.2022.757084 (PMC8882863; doi:10.3389/fmicb.2022.757084)
Supplement: Supplementary file 1 [file Data_Sheet_1.pdf]

Supplementary Material

Biological Characterization of Yellow Fever Viruses Isolated From Non-human Primates in Brazil With Distinct Genomic Landscapes

Nathália Dias Furtado[1], Lidiane de Menezes Raphael[1], Ieda Pereira Ribeiro[1],Iasmim Silva de Mello[1], Déberli Ruiz Fernandes[1], Mariela Martínez Gómez[2], Alexandre Araújo Cunha dos Santos[1], Mônica da Silva Nogueira[3], Márcia Gonçalves de Castro[4], Filipe Vieira Santos de Abreu[4], Lívia Carício Martins[5], Pedro Fernando da Costa Vasconcelos[5], Ricardo Lourenço-de-Oliveira[4] and Myrna Cristina Bonaldo[1]*

[1]Laboratório de Biologia Molecular de Flavivírus, Instituto Oswaldo Cruz/FIOCRUZ, Rio de Janeiro, Brazil, [2]Instituto de Investigaciones Biológicas Clemente Estable, Montevideo, Uruguay, [3]Centro de Experimentação Animal, Instituto Oswaldo Cruz/FIOCRUZ, Rio de Janeiro, Brazil, [4]Laboratório de Mosquitos Transmissores de Hematozoários, Instituto Oswaldo Cruz/FIOCRUZ, Rio de Janeiro, Brazil, [5]Seção de Arbovirologia e Febres Hemorrágicas, Instituto Evandro Chagas/FIOCRUZ, Pará, Brazil

Supplementary Table 1 - Variable sites between the YFV isolates.

	Genome position	YFV 2000 - 2010		YFV 2016 - 2019		Codon change	Amino acid variation
		GO05	PR4408	ES-504	RJ 155		
**5'UTR**	53	**A**	G	G	G	-	-
**CDS**	217	A	**G**	A	A	CAA - CAG	-
	350	T	**C**	T	T	TTA - CTA	-
	426	A	A	A	**G**	CAA - CGA	Q - R
	436	A	A	G	G	TTG - TTA	-
	440	G	G	A	A	ATA - GTA	I - V
	451	G	G	A	A	TTA - TTG	-
	499	**G**	A	A	A	AGA - AGG	-
	553	T	T	C	C	TCC - TCT	-
	598	T	T	C	C	TAC - TAT	-
	784	**G**	A	A	A	TTA - TTG	-
	841	A	**G**	A	A	AAA - AAG	-
	973	G	**A**	G	G	TCG - TCA	-
	982	**C**	T	T	T	TGT - TGC	-
	997	C	C	C	**T**	GAC - GAT	-
	1030	**T**	C	C	C	ACC - ACT	-
	1045	C	**T**	C	C	ACC - ACT	-
	1054	G	G	G	**A**	CAG - CAA	-

	Genome position	YFV 2000 - 2010		YFV 2016 - 2019		Codon change	Amino acid variation
		GO05	PR4408	ES-504	RJ 155		
**CDS**	1063	T	**C**	T	T	TGT - TGC	-
	1168	A	A	G	G	TTG - TTA	-
	1342	A	A	A	**G**	AAA - AAG	-
	1426	T	T	C	C	AAC - AAT	-
	1468	A	A	G	G	TCG - TCA	-
	1480	**G**	A	A	A	GAA - GAG	-
	1507	**T**	C	C	C	GCC - GCT	-
	1525	A	A	G	G	GTG - GTA	-
	1543	**T**	C	C	C	TTC - TTT	-
	1546	C	Y	T	T	AGT - AGC	-
	1549	**T**	C	C	C	AAC - AAT	-
	1618	**G**	A	A	A	CTA - CTG	-
	1621	T	**C**	T	T	CCT - CCC	-
	1636	**C**	T	T	T	AGT - AGC	-
	1690	T	T	C	C	GCC -GCT	-
	1696	C	C	T	T	ACT - ACC	-
	1720	C	**T**	C	C	AAC - AAT	-
	1780	T	T	C	C	AAC - AAT	-

	Genome position	YFV 2000 - 2010		YFV 2016 - 2019		Codon change	Amino acid variation
		GO05	PR4408	ES-504	RJ 155		
**CDS**	1813	C	C	T	T	CAT - CAC	-
	1849	**C**	T	T	T	CTT - CTC	-
	1864	**T**	C	C	C	TAC - TAT	-
	1909	**T**	C	C	C	GAC - GAT	-
	1921	**T**	C	C	C	GGC - GGT	-
	1924	T	**C**	T	T	ACT - ACC	-
	2047	**T**	C	C	C	ACC - ACT	-
	2119	**C**	T	T	T	GAT - GAC	-
	2128	G	**A**	G	G	CTG - CTA	-
	2188	G	**A**	G	G	AAG - AAA	-
	2200	C	C	T	T	CGT - CGC	-
	2215	G	G	A	A	GGA - GGG	-
	2224	**T**	C	C	C	GCC - GCT	-
	2251	C	**T**	C	C	TTC -TTT	-
	2306	C	**T**	C	C	CTG - TTG	-
	2350	**G**	A	A	A	GTA - GTG	-
	2434	**T**	G	G	G	CTG - CTT	-
	2500	**A**	G	G	G	GGG - GGA	-

	Genome position	YFV 2000 - 2010		YFV 2016 - 2019		Codon change	Amino acid variation
		GO05	PR4408	ES-504	RJ 155		
CDS	2584	G	A	A	A	TCA - TCG	-
	2605	G	G	G	T	GAG - GAT	E - D
	2611	A	A	G	G	GGG - GGA	-
	2659	G	A	A	A	AGA - AGG	-
	2755	G	A	A	A	ACA - ACG	-
	2827	T	T	C	C	TCC - TCT	-
	2887	T	T	C	C	TTC - TTT	-
	2974	A	A	G	G	GAG - GAA	-
	3007	G	T	T	T	GGT - GGG	-
	3037	C	T	T	T	CAT - CAC	-
	3097	C	C	T	T	ACC - ACT	-
	3134	C	C	T	C	CTG - TTG	-
	3187	A	G	G	G	AGG - AGA	-
	3232	C	T	T	T	TAT - TAC	-
	3303	G	A	G	G	AGT - AAT	S - N
	3446	C	T	C	C	CCT - TCT	P - S
	3502	G	G	A	A	GTG - GTA	-
	3568	A	G	A	A	GTA - GTG	-

	Genome position	YFV 2000 - 2010		YFV 2016 - 2019		Codon change	Amino acid variation
		GO05	PR4408	ES-504	RJ 155		
CDS	3607	A	A	G	G	GGG - GGA	-
	3622	**G**	A	A	A	CTA - CTG	-
	3632	T	T	C	C	CTA - TTA	-
	3686	C	**T**	C	C	CTG - TTG	-
	3725	**C**	T	T	T	TAT - CAT	Y - H
	3739	C	C	C	**T**	ATC - ATT	-
	3764	**T**	C	C	C	CTG - TTG	-
	3895	A	A	G	G	GTG - GTA	-
	3907	**T**	C	C	C	ATC - ATT	-
	3910	**A**	G	G	G	CTG - CTA	-
	3919	T	T	C	C	AAC - AAT	-
	3973	G	G	A	A	TTA - TTG	-
	4030	T	T	C	C	GTC - GTT	-
	4099	**A**	**T**	C	C	CTC - CTA/T	-
	4103	**T**	C	C	C	CTG - TTG	-
	4111	T	T	C	C	TCC - TCT	-
	4198	**G**	A	A	A	GAA - GAG	-
	4229	T	**C**	T	T	TTG - CTG	-

	Genome position	YFV 2000 - 2010		YFV 2016 - 2019		Codon change	Amino acid variation
		GO05	PR4408	ES-504	RJ 155		
**CDS**	4243	**C**	T	T	T	GCT - GCC	-
	4267	**G**	A	A	A	CTA - CTG	-
	4276	C	C	T	T	GTT - GTC	-
	4330	**G**	A	A	A	GGA - GGG	-
	4372	**G**	A	A	A	GAA - GAG	-
	4408	G	G	A	A	GTA - GTG	-
	4504	T	T	C	C	GCC - GCT	-
	4522	C	C	T	T	CTT - CTC	-
	4525	**G**	A	A	A	TTA - TTG	-
	4591	T	T	C	C	GAC - GAT	-
	4654	T	T	C	C	TTC - TTT	-
	4660	**T**	A	A	A	TCA - TCT	-
	4669	A	**G**	A	A	CTA - CTG	-
	4717	C	C	T	T	CAT - CAC	-
	4759	C	C	T	T	AAT - AAC	-
	4769	C	**T**	C	C	CTG - TTG	-
	4834	G	G	T	T	GAT - GAG	D - E
	4858	**A**	G	G	G	GAG - GAA	-

	Genome position	YFV 2000 - 2010		YFV 2016 - 2019		Codon change	Amino acid variation
		GO05	PR4408	ES-504	RJ 155		
**CDS**	4882	**G**	C	C	C	CCC - CCG	-
	4932	G	G	A	A	AAG - AGG	K - R
	4948	C	C	T	T	ATC - ATT	-
	4972	G	G	A	A	CCA - CCG	-
	4993	C	**T**	C	C	CCC - CCT	-
	5053	**T**	C	C	C	GAC - GAT	-
	5115	A	**G**	A	A	CAG - CGG	Q - R
	5152	C	**T**	C	C	ACC - ACT	-
	5185	A	**G**	A	A	ACA - ACG	-
	5200	T	**C**	T	T	CCT - CCC	-
	5239	A	**C**	A	A	ACA - ACC	-
	5242	**C**	T	T	T	CTT - CTC	-
	5251	T	**A**	T	T	GCT - GCA	-
	5254	C	C	T	T	CCT - CCC	-
	5269	C	C	T	T	CTT - CTC	-
	5296	C	C	T	T	GGT - GGC	-
	5335	C	C	C	**T**	CAC - CAT	-
	5349	A	A	A	**G**	GAG - GGG	E - G

	Genome position	YFV 2000 - 2010		YFV 2016 - 2019		Codon change	Amino acid variation
		GO05	PR4408	ES-504	RJ 155		
CDS	5350	A	A	G	G	GAG - GAA	-
	5362	C	**T**	C	C	GCC - GCT	-
	5383	A	**G**	A	A	ACA - ACG	-
	5452	A	A	G	G	TTG - TTA	-
	5473	**T**	C	C	C	GCC - GCT	-
	5479	C	C	C	**T**	GGC - GGT	-
	5512	**C**	T	T	T	AGT - AGC	-
	5521	C	**T**	C	C	ATC - ATT	-
	5533	C	C	T	T	GCT - GCC	-
	5605	**C**	T	T	T	CCT - CCC	-
	5614	T	T	T	**C**	CCT - CCC	-
	5713	C	C	C	**T**	CGC - CGT	-
	5992	C	C	T	T	GAT - GAC	-
	6031	A	A	G	G	GCG - GCA	-
	6043	**T**	C	C	C	TGC - TGT	-
	6055	**T**	C	C	C	GCC - GCT	-
	6109	**C**	T	T	T	TAT - TAC	-
	6139	**T**	C	C	C	TCC - TCT	-

	Genome position	YFV 2000 - 2010		YFV 2016 - 2019		Codon change	Amino acid variation
		GO05	PR4408	ES-504	RJ 155		
**CDS**	6148	**G**	A	A	A	GAA - GAG	-
	6199	C	C	T	T	AAT - AAC	-
	6229	A	**G**	A	A	CAA - CAG	-
	6325	**A**	G	G	G	AAG - AAA	-
	6328	**T**	C	C	C	TGC - TGT	-
	6607	G	**A**	G	G	CTG - CTA	-
	6652	C	**T**	C	C	TTC - TTT	-
	6682	**C**	T	T	T	TCT - TCC	-
	6766	G	G	A	A	CTA - CTG	-
	6835	G	G	A	A	CAA - CAG	-
	6850	C	C	T	T	ATT - ATC	-
	6893	C	**T**	C	C	CTA - TTA	-
	6934	A	A	A	**G**	AAA - AAG	-
	6961	**C**	A	A	A	GCA - GCC	-
	7075	**C**	T	T	T	GGT - GGC	-
	7144	**C**	T	T	T	TTT - TTC	-
	7183	**C**	T	T	T	AGT - AGC	-
	7240	T	T	A	A	CTA - CTT	-

	Genome position	YFV 2000 - 2010		YFV 2016 - 2019		Codon change	Amino acid variation
		GO05	PR4408	ES-504	RJ 155		
**CDS**	7397	**C**	T	T	T	TTG - CTG	-
	7423	T	**C**	T	T	CTT - CTC	-
	7453	**G**	A	A	A	ACA - ACG	-
	7459	T	T	C	C	TTC - TTT	-
	7504	**A**	G	G	G	CCG - CCA	-
	7597	**G**	T	T	T	GGT - GGG	-
	7600	C	C	T	T	GTT - GTC	-
	7684	G	**A**	G	G	CTG - CTA	-
	7688	C	**T**	C	C	CTG - TTG	-
	7738	A	A	G	G	GAG - GAA	-
	7756	T	**C**	T	T	GCT - GCC	-
	7837	**G**	A	A	A	GAA - GAG	-
	7869	**T**	C	C	C	ACT - ATT	T - I
	7920	G	**A**	G	G	AGA - AAA	R - K
	7936	T	Y	C	C	GTC - GTT	-
	7938	A	A	G	G	AGG - AAG	R - K
	7972	G	**A**	G	G	AAG - AAA	-
	8006	**G**	A	A	A	ATC - GTC	I - V

	Genome position	YFV 2000 - 2010		YFV 2016 - 2019		Codon change	Amino acid variation
		GO05	PR4408	ES-504	RJ 155		
**CDS**	8038	**T**	C	C	C	CGC - CGT	-
	8047	A	A	G	G	CCG - CCA	-
	8048	G	G	A	A	ATA - GTG	I - V
	8050	G	G	A	A		
	8059	T	**C**	T	T	GAT - GAC	-
	8152	G	**A**	G	G	TTG - TTA	-
	8153	G	G	A	A	AGC - GGC	S - G
	8161	C	C	T	T	GGT - GGC	-
	8221	**G**	A	A	A	TTA - TTG	-
	8386	A	A	G	G	AAG - AAA	-
	8407	T	T	C	C	GTC - GTT	-
	8443	T	**C**	T	T	GAT - GAC	-
	8494	G	A	A	G	AAA - AAG	-
	8497	T	T	A	A	TCA - TCT	-
	8526	A	A	G	G	AGT - AAT	S - N
	8614	C	C	C	**T**	ATC - ATT	-
	8623	**A**	G	G	G	TTG - TTA	-
	8734	T	T	T	**C**	GAT - GAC	-

	Genome position	YFV 2000 - 2010		YFV 2016 - 2019		Codon change	Amino acid variation
		GO05	PR4408	ES-504	RJ 155		
**CDS**	8773	**C**	T	T	T	AAT - AAC	-
	8809	C	C	C	**A**	AAC - AAA	N - K
	8821	T	T	C	C	TGC - TGT	-
	8851	C	C	C	**A**	CGC - CGA	-
	9082	C	**T**	C	C	GCC - GCT	-
	9210	**T**	C	C	C	GCA - GTA	A - V
	9277	**C**	T	T	T	GAT - GAC	-
	9322	T	**C**	T	T	CAT - CAC	-
	9334	G	**A**	G	G	GCG - GCA	-
	9343	**G**	A	A	A	GTA - GTG	-
	9418	C	**T**	C	C	GTC - GTT	-
	9445	T	**C**	T	T	TCT - TCC	-
	9463	**T**	C	C	C	TAC - TAT	-
	9502	T	T	T	**G**	ATG - ATT	I - M
	9517	**G**	A	A	A	GCA - GCG	-
	9547	G	**A**	G	G	CAG - CAA	-
	9564	T	T	C	C	GCT - GTT	A - V
	9570	C	C	C	**T**	ACC - ATC	T - I

	Genome position	YFV 2000 - 2010		YFV 2016 - 2019		Codon change	Amino acid variation
		GO05	PR4408	ES-504	RJ 155		
CDS	9592	**C**	T	T	T	GCT - GCC	-
	9601	C	**T**	C	C	GGC - GGT	-
	9613	C	C	T	T	CTT - CTC	-
	9685	C	**T**	C	C	TCC - TCT	-
	9691	T	T	C	C	CTC - CTT	-
	9715	**G**	A	A	A	AAA - AAG	-
	9727	A	**G**	A	A	GAA - GAG	-
	9762	A	A	G	G	AGT - AAT	S - N
	9772	C	C	T	T	TTT - TTC	-
	9794	C	**T**	C	C	CTG - TTG	-
	9800	C	C	T	T	TTG - CTG	-
	9838	**C**	T	T	T	GAT - GAC	-
	9859	A	A	G	G	AGG - AGA	-
	9877	A	A	G	G	GGG - GGA	-
	9883	T	T	T	**C**	GGT - GGC	-
	10039	**C**	T	T	T	GTT - GTC	-
	10151	G	**A**	G	G	GAC - AAC	D - N
	10171	**A**	G	G	G	AAG - AAA	-

Region	Genome position	YFV 2000 - 2010		YFV 2016 - 2019		Codon change	Amino acid variation
		GO05	PR4408	ES-504	RJ 155		
**CDS**	10210	C	**T**	C	C	ACC - ACT	-
	10222	T	**C**	T	T	ACT - ACC	-
	10255	**T**	C	C	C	CGC - CGT	-
**3'UTR**	10374	**C**	T	T	T	-	-
	10488	G	G	**A**	G	-	-
	10544	T	T	C	C	-	-
	10559	**C**	T	T	T	-	-
	10612	**T**	C	C	C	-	-
	10709	**C**	T	T	T	-	-
	10750	**T**	C	C	C	-	-
	10751	T	T	C	C	-	-
	10764	**G**	A	A	A	-	-
	10883	C	C	T	T	-	-
	10990	C	**T**	C	C	-	-
	10993	A	**C**	A	A	-	-

		Variable sites	%
**UTR**		13	5.2%
**CDS**	Synonymous	211	84.7%
	Non-synonymous	25	10.0%
	Total:	249	100%

Supplementary Table 2 - Clinical assessment and determination of humane endpoint.

Observation	Score
**Body weight**	
Loss of 5-10%	1
Loss of 10-15%	2
Loss of 16-20%	3
Loss of 20% or more	EUTHANASIA
**Fur**	
Slightly ruffled	2
Evidently ruffled	3
**Respiration**	
Accelerated breathing (tachypnea)	1
Difficult breathing (dyspnea)	3
**Clinical complications**	
Tension during handling	1
Tremors, breath noises, aggression, vocalizations	3
Repetitive movements indicating neurological impairment	EUTHANASIA
**Motility**	
Abnormal walking and posture (slightly hunched, reduced activity)	1
Massively abnormal walking and posture (evidently hunched, moderately reduced activity)	2
Motility only after stimulation, isolation, lethargy	3
No activity for more than 24 hours	EUTHANASIA

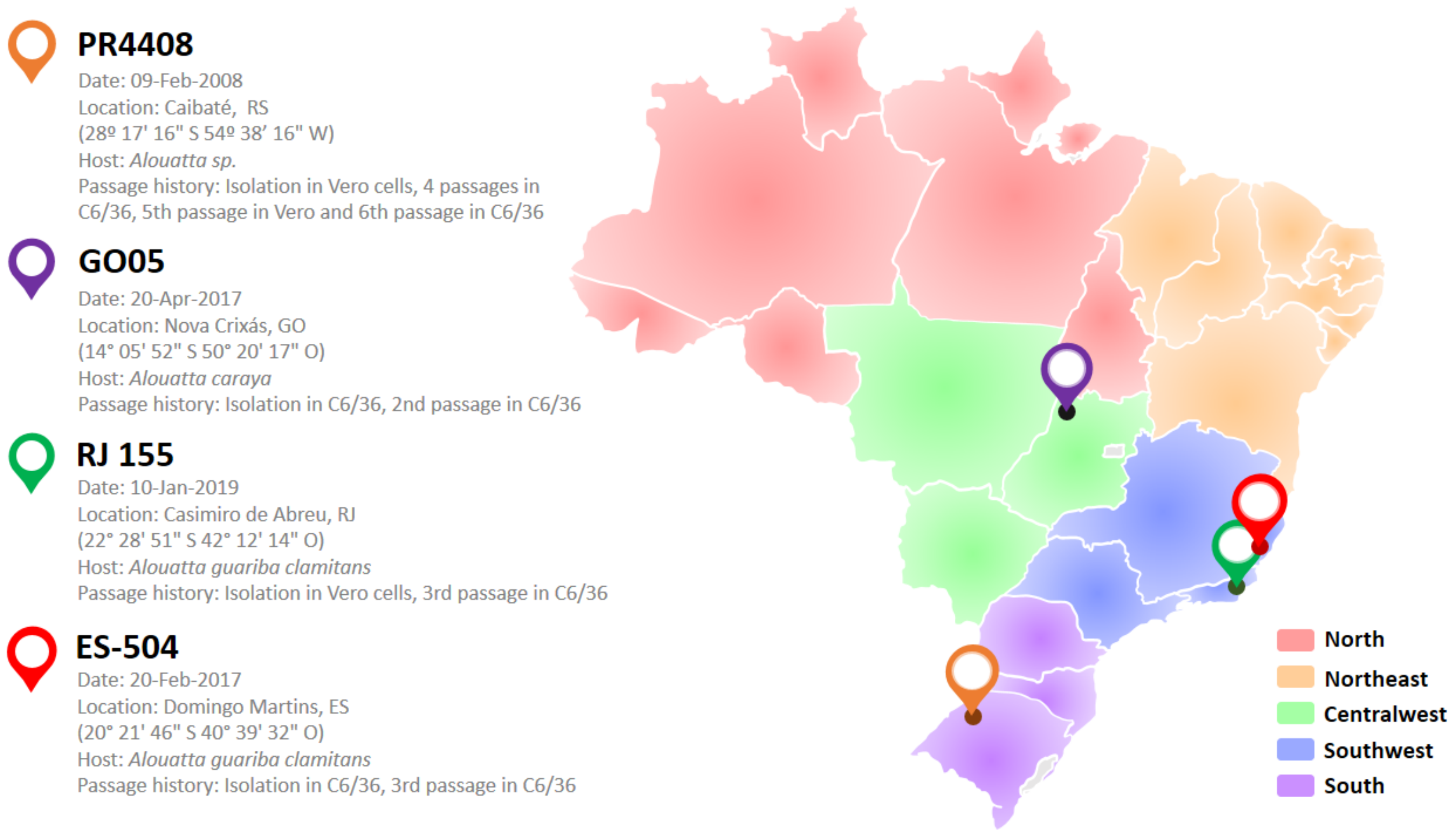

Supplementary Figure 1 - YFV isolates information. Geographic map of Brazil depicted by region. Information about the date of collection, host and cell passage history is described for each YFV isolate, and colored pins indicate their location.

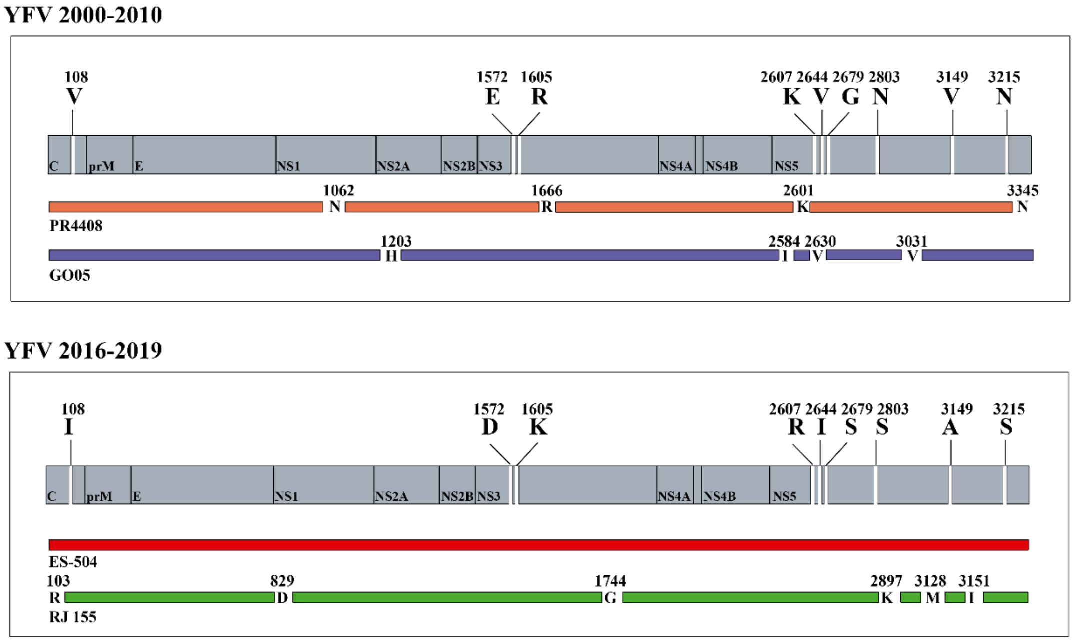

Supplementary Figure 2 - Schematic representation of the four isolates' genomic composition. The viral precursor polyprotein is represented by the rectangles, and the molecular signature that differentiates the groups is described for each group. The unique amino acid changes of each isolate are depicted in the respective representative rectangle.

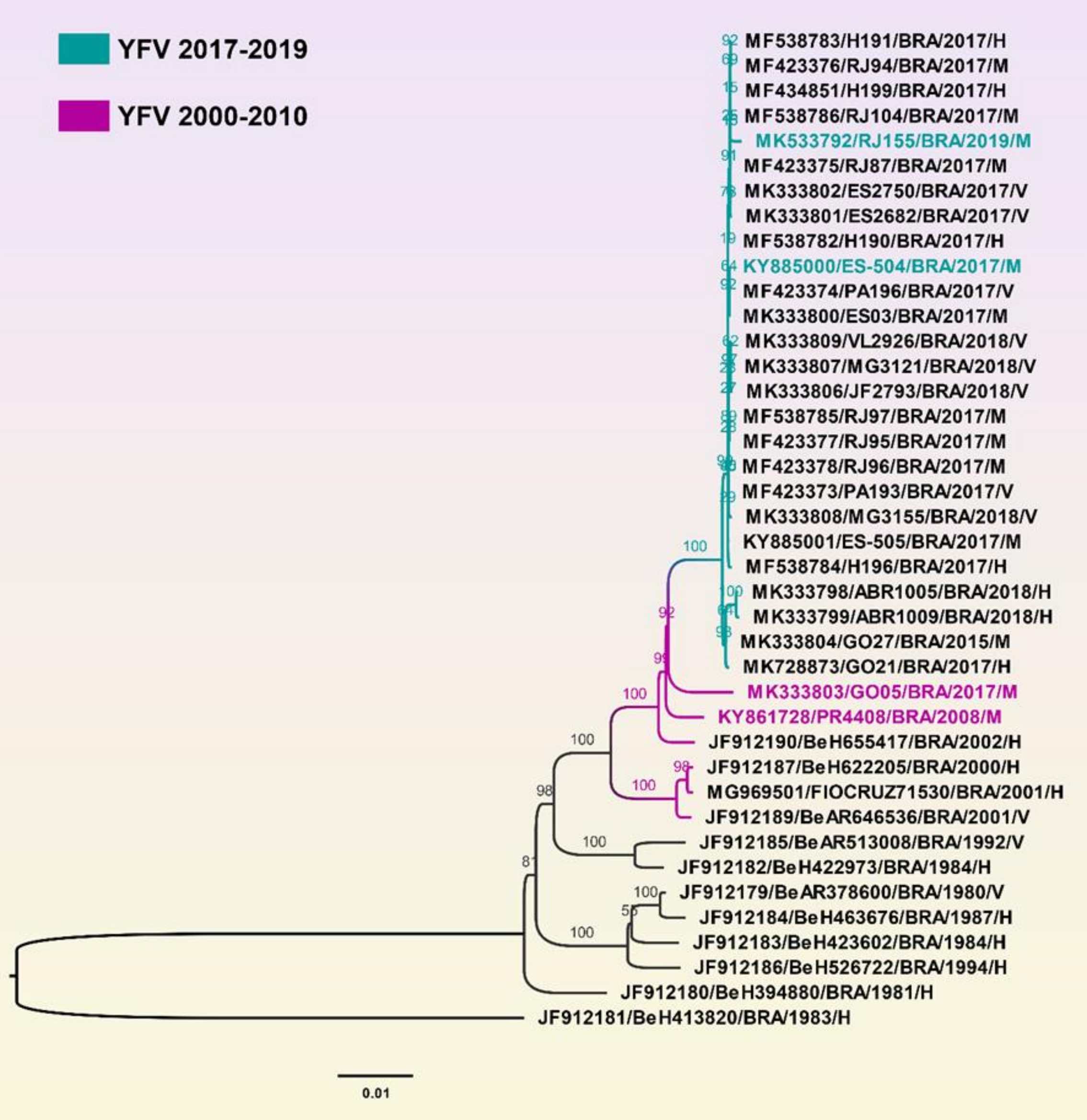

Supplementary Figure 3 - Phylogenetic analysis of Brazilian YFV complete genomes. YFV complete genome records were retrieved from GenBank database and aligned using AliView. The Maximum Likelihood analysis was carried out with 1000 bootstrap replicates. The sequences that carry the molecular signature of YFV 2016-2019 are depicted in green and the other sequences belonging to modern lineage 1E are depicted in purple (YFV 2000-2010).

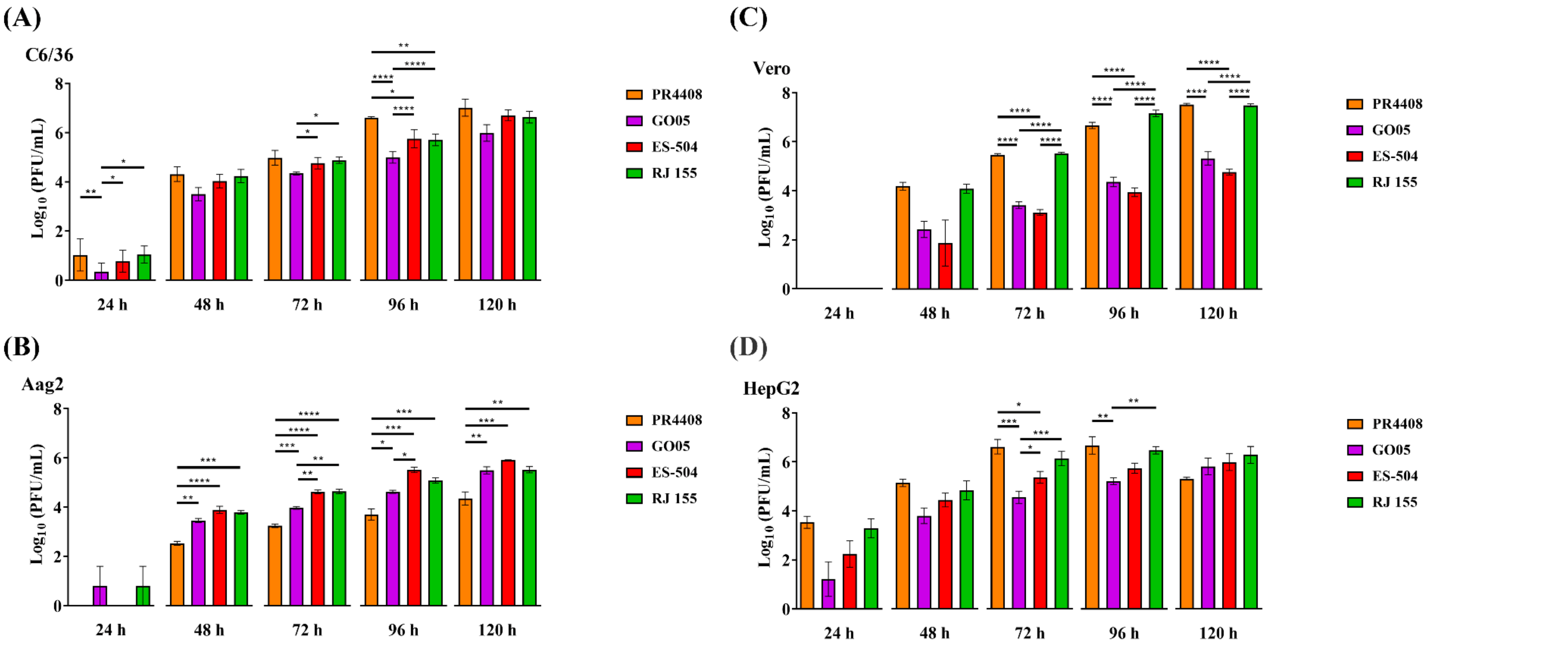

Supplementary Figure 4 - Statistical analyses of viral growth over time in C6/36 cells (A), Aag2 (B), Vero (C) and HepG2 (D). Viral titers average of each YFV isolate were compared using One-way ANOVA with Bonferroni's multiple comparisons test: * represents $P \leq 0.05$, ** represents $P \leq 0.01$, *** represents $P \leq 0.001$ and **** represents $P \leq 0.0001$.

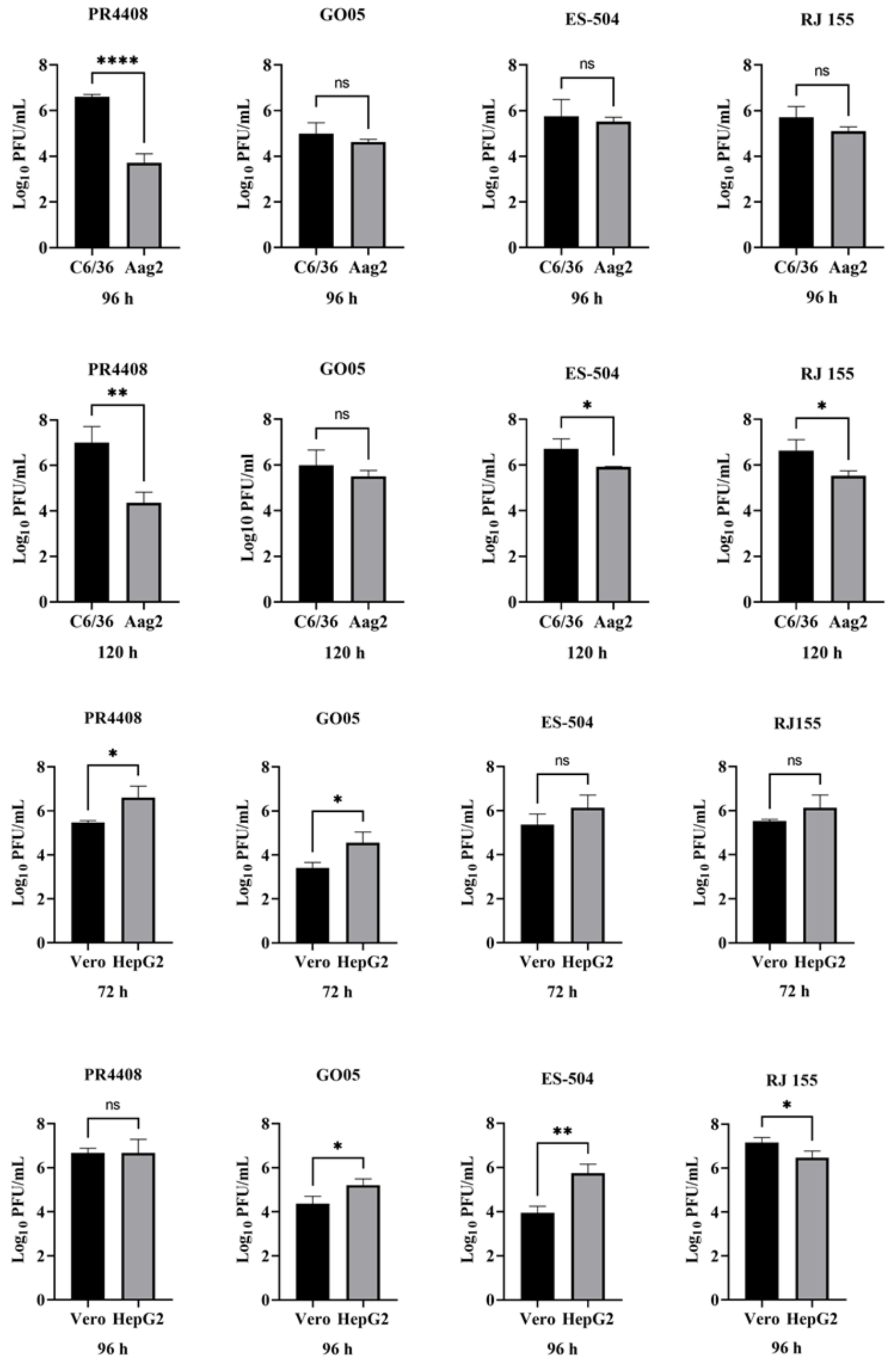

Supplementary Figure 5 - Comparison of viral growth between the cell lines. Statistical analysis of viral growth peaks for each isolate in mosquito cells and mammal cells. Viral titers averages were compared using Unpaired t test: "ns" means not significant, * represents $P \leq 0.05$, ** represents $P \leq 0.01$ and **** represents $P \leq 0.0001$.

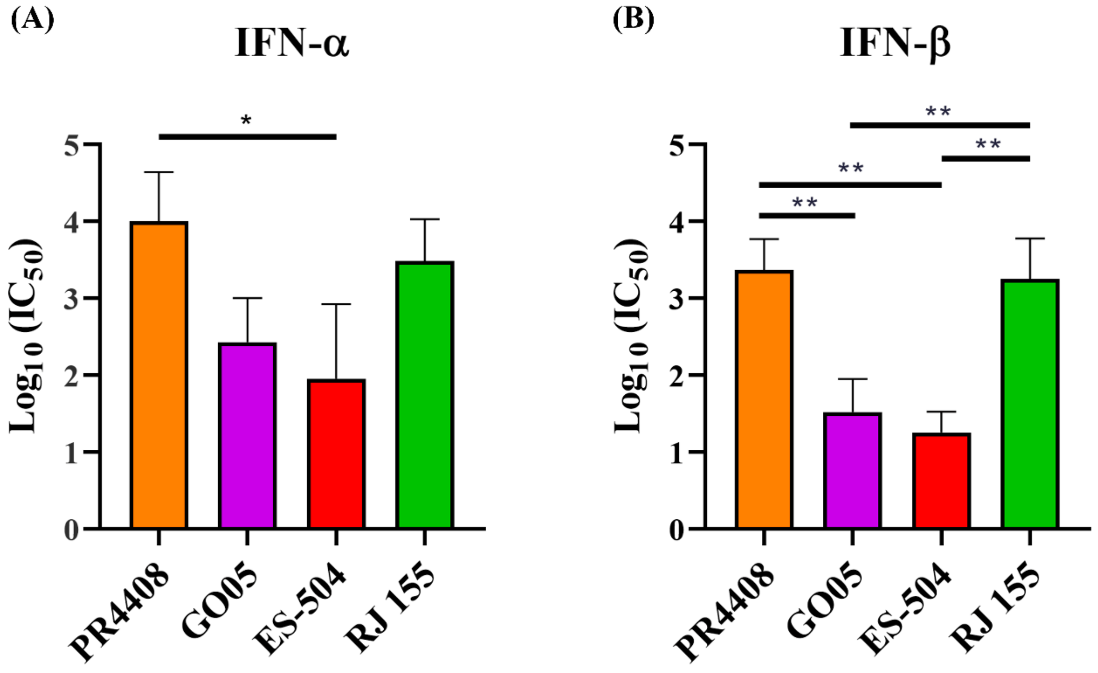

Supplementary Figure 6 - Statistical analysis of IC_{50} values obtained from viral growth in the presence of type I interferon. The IC_{50} values of each replicate were plotted in a column table and analyzed for statistical differences. The test applied was One-way ANOVA with Bonferroni's multiple comparisons test: * represents $P \leq 0.05$ and ** represents $P \leq 0.01$.
